# *BCR-ABL1* is a secondary event after JAK2V617F in a patient with essential thrombocythemia who develop chronic myeloid leukemia

**DOI:** 10.1097/BS9.0000000000000129

**Published:** 2022-08-01

**Authors:** Yanqing Zhang, Hailiang Bi, Ying Wang, Long Chen, Jiaqi Pan, Ping Xu, Wei Wang, Shaobin Yang

**Affiliations:** a Department of Hematology, The Second Affiliated Hospital of Harbin Medical University, Harbin, Heilongjiang, P. R. China; b The Seventh Affiliated Hospital, Sun Yat-sen of University, Shenzhen, Guangdong, P. R. China; c Department of Molecular Biology Laboratory, Tianjin SINO-US-Diagnostics Co. Ltd, Tianjin, P. R. China

**Keywords:** BCR-ABL1, Chronic myeloid leukemia, Clonal evolution, JAK2 V617F, Postessential thrombocythemia myelofibrosis

## Abstract

Several cases such as myeloproliferative neoplasms (MPN) with the coexistence of JAK2 and BCR-ABL have been reported. However, cases of transformation of essential thrombocythemia (ET) into chronic myeloid leukemia (CML) during the disease progression were rarely reported. Here, we report the case of a patient with *JAK2 V617F*- positive ET who subsequently acquired BCR–ABL1, which transformed the disease into CML after 10 years from the initial diagnosis. In this study, we dynamically monitored JAK2 V617F and BCR-ABL and observed multiple gene mutations, including IDH2, IDH1, ASXL1, KRAS, and RUNX1. It is important to be aware of this potentially clone evolution in disease progression.

## 1. INTRODUCTION

Myeloproliferative neoplasms (MPN) are caused by hematopoietic stem cells (HSCs) with somatic mutations in the genes involved in the tyrosine kinase signaling. The main affected genes include the BCR-ABL1 in Philadelphia chromosome-positive chronic myeloid leukemia (CML) and JAK2/MPL/CALR mutations in MPNs. Although it was thought to be mutually exclusive, a number of cases with coexistence of JAK2 V617F mutation and BCR-ABL in patient with MPN have been reported.^[1-4]^ However, the cases of transformation of essential thrombocythemia (ET) into CML during the disease progression were rarely reported. Here, we report the case of a patient with JAK2 V617F-positive ET who subsequently acquired BCR-ABL1 that transformed into CML after 10 years from the initial diagnosis. In this study, we dynamically monitored clinical variables, hematologic data, bone marrow (BM) histomorphologic features, karyotype, JAK2 V617F, and BCR-ABL and observed multiple gene mutations by next-generation sequencing (NGS), including IDH2, IDH1, ASXL1, KRAS, RUNX1 etc. Although the case was rare, it is important to be aware of this potential clone evolution during disease progression. These features can be misinterpreted to reflect resistance to therapy or disease progression.

## 2. CASE PRESENTATION

Clinical characteristics, laboratory results, BM biopsy results, response, and prognosis of a case of a 48-year-old-male patient with ET who developed CML after 10 years from the initial diagnosis in The Second Affiliated Hospital of Harbin Medical University were retrospectively collected and analyzed. A 48-year-old-male patient was diagnosed with JAK2V617F-positive ET, normal cytogenetics, and absence of BCR-ABL 10 years ago (2010). He was treated with hydroxyurea or interferon-alpha (IFN-α) until March 2017. A routine blood monitoring showed that white blood cell (WBC) was between 5 and 10 × 10^9^/L, and the platelet (PLT) was between 400 and 600 × 10^9^/L. He had a history of coronary stent implantation and irregularly followed oral administration of antiplatelet aggregation drugs and statins in 2017. In the same year (2017), the patient suffered from right lower abdominal pain and his blood routine examination revealed high WBC:26 × 10^9^/L. Thus, he was diagnosed with acute appendicitis and took anti-inflammatory medication and appendectomy. After surgery, the abdominal pain improved, but WBC was still higher than normal. At that time, the patient did not pay attention to it. Routine blood monitoring showed that the WBC was between 15 and 30 × 10^9^/L, and the PLT was between 100 and 300 × 10^9^/L. The patient continued to take hydroxyurea treatment.

**Figure 1.**
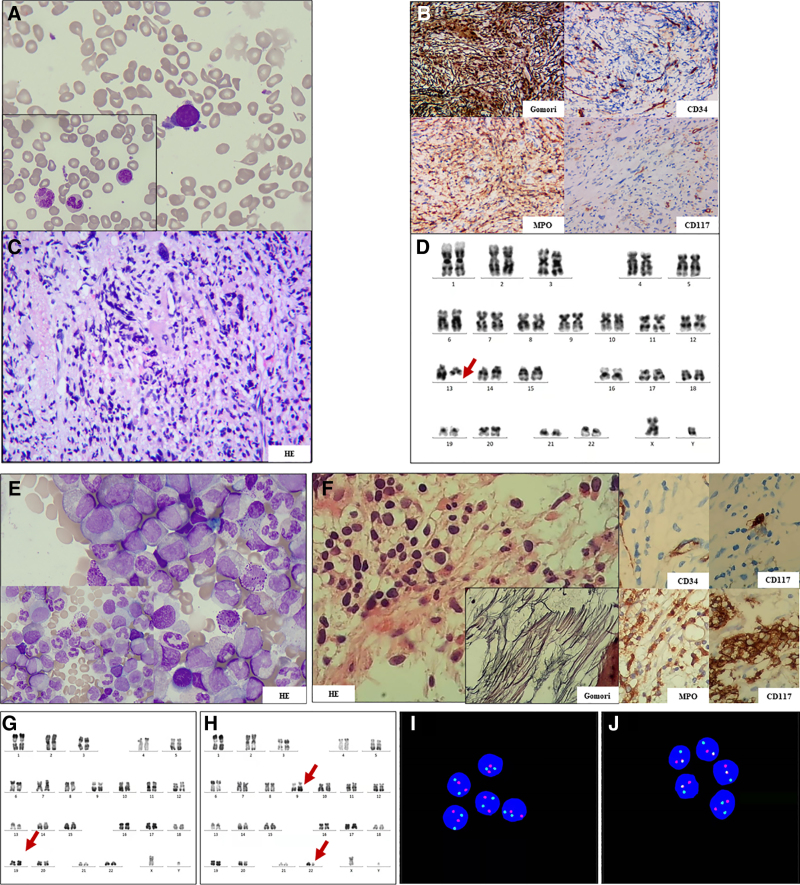
Morphology and cytogenetic analysis. (A) Bone marrow (BM) morphology at post-ET MF diagnosis, wright stained, ×100 magnification. (B and C) BM biopsy analysis at post-ET MF diagnosis, hematoxylin and eosin stained, ×40 magnification. (D) Karyotype at post-ET MF diagnosis: 46, XY, del(13)(q13q21). (E) BM morphology at CML diagnosis, wright stained, ×100 magnification. (F) BM biopsy analysis at CML diagnosis, hematoxylin and eosin stained, ×40 magnification. (G and H) Karyotype at CML diagnosis: 46, XY, t(9;22)(q34;q11) and 46, XY, del(13)(q13q21). (I and J) Interphase fluorescence in situ hybridization (iFISH) (Vysis dual color, dual-fusion translocation probe) analysis of *BCR::ABL1* (*ABL1* red, *BCR* green). Abbreviation: CML = chronic myeloid leukemia.

However, in October 2020, the patient had splenomegaly and hepatosplenomegaly was clinically confirmed by ultrasound (spleen length: 16.5 mm). Full blood count revealed a WBC of 41.97 × 10^9^/L, HB of 128 g/L, PLT of 307 × 10^9^/L, and an absolute neutrophil count of 30.38 × 10^9^/L. BM aspirate was mildly hypercellular with increased megakaryopoiesis and a normal megakaryocyte:erythrocyte (M:E) ratio (Fig. 1A). A BM biopsy examination showed a hypercellular marrow and predominant megakaryocytic proliferation, with very large and polyploid megakaryocytes arranged in tight clusters. Megakaryocytic proliferation was associated with a marked myeloid hyperplasia and the reticulin stain showed increased fibrosis (grade 3) (Fig. 1B and C). Cytogenetic analysis of the BM was abnormal (46, XY, del(13)(q13q21)[10]/ 46,XY[10]) when assessing twenty metaphases (Fig. 1D). In addition, we detected several mutations by next-generation sequencing (NGS). Nucleated cells (1.0 × 10^7^) were used for genomic DNA extraction after the lysis of red blood cells. NGS of genomic DNA was performed on the coding sequence (CDS) of the 175 hematological disease genes through Illumina NextSeq 550 with a mean sequencing depth of 2000 ◊. Data were analyzed using the bioinformatics pipeline in house (Data S1). The results found gene mutations, including JAK2 c.1849G>T/p.V617F (90.20%), IDH2 c.419G>A/p.R14OQ (44.90%), ASXL1 c.1772dupA/p.Y519*fs*1(12.10%), KRAS c.190T>G/p.Y64D (5.80%), RUNX1 c.508C>G/p.P203R (4.50%), and IDH1 c.395G>A/p.R132H(1.90%) (Data S1). Thus, the patient was diagnosed with post-ET myelofibrosis (post-ET MF) and treated with ruxolitinib (20 mg twice a day). The patient’s WBCs did not decrease with the intake of ruxolitinib and remained between 40 and 50 × 10^9^/L, and the spleen did not retract. The patient continued and persisted with the medication for 1 year. However, in September 2021, a physical examination of the patient during treatment with ruxolitinib showed a rapid and massive splenomegaly (19 cm below costal margin) and hepatomegaly (3 cm below costal margin). In addition, leukocytosis (WBC = 143 × 10^9^/L) and peripheral blood examination showed a mild anemia and a marked leukocytosis with a neutrophilic left shift, a significantly increased number of basophils (27%) and rare blasts (5%) (Table 1). BM aspirate and biopsy showed typical features of CML with granulocytic and megakaryocytic hyperplasia, and fibrosis, no excess blasts (grade 3) on reticulin staining (Fig. 1E and F). Cytogenetic analysis revealed the presence of a Philadelphia chromosome translocation t(9;22)(q34;q11)[28/30]. In addition, there was an abnormal chromosome del(13)(q13q21)[1/30] (Fig. 1G an H). Fluorescence in situ hybridization (FISH) of BM showed typical *BCR::ABL1* dual-fusion signals in in 92% (460/500) (Fig. 1I and J). Molecular analysis confirmed the presence of a BCR-ABL fusion (b3a2). Real-time quantitative (RTQ)-PCR showed *BCR::ABL1* was 102.22% and JAK2 mutant allele burden in bone marrow cells as 91.38%. A diagnosis of accelerated-phase (AP) CML was made, and the patient was started on the tyrosine kinase inhibitor (TKI) imatinib in September 2021. Imatinib therapy was ceased 1 month later as a result of the intolerable therapy, and the patient was started on fumatinib and ruxolitinib. After 3 months of therapy, which produced a hematologic response only, the JAK2V617F allele burden was 91.76%, but BCR-ABL was 38.31%. The patient was indicated splenomegaly (17 cm below costal margin). At the last follow-up in May 2022, WBC of the patient had a significant decrease, 20.4 × 10^9^/L; hemoglobin 99 g/L, and platelets 83 × 10^9^/L; the JAK2 V617F allele burden was 92.54%, but BCR-ABL was 0.24%; and the spleen was decreased to 13 cm below the costal surface. The next treatment will consider radiotherapy on the spleen and HSC transplantation (HSCT).

**Table 1:** Clinical characteristics of the patient in three phases.

Characteristics	ET phase	Post-ET MF phase	CML phase
Size of spleen (below costal margin, cm)	Normal	11	15
Hemoglobin, g/dL	132	128	92
Platelet count, ×10^9^/ L	658	307	135
WBC count, ×10^9^/ L	7.2	41.97	128.6
Neutrophils absolute count, ×10^9^/ L (%)	3.8	37.35	36
Blasts (%)	0	2	5
Promyelocytes (%)	0	0	7
Myelocyts (%)	0	2	3
Metamyelocytes (%)	0	1	11
Bands (%)	0	2	10
Neutrophils (%)	75	83.1	28
Lymphocytes (%)	20	2.9	1
Monocytes (%)	4	3.1	3
Eosinophils (%)	0	1.4	3
Basophils (%)	1	2.5	29
LDH, IU/L	189	661	1233
Karyotype (ISCN)	46, XY[20]	46,XY,del(13)(q12q22)[12]/46, XY[8]	46,XY,t(9;22)(q34;q11.2)[28]/46,XY,del(13)(q13q21)[1]/46,XY[1]
FISH BCR-ABL	0	0.50%	92.50%
JAK2 V617F (PCR)	Positive	Positive	96.2

Abbreviations: CML = chronic myeloid leukemia, ET = essential thrombocythemia, FISH = fluorescence in situ hybridization, LDH = lactate dehydrogenase, MF = myelofibrosis, WBC = white blood cell.

## 3. DISCUSSION

We reported a case of a patient who was previously diagnosed with ET and treated with hydroxyurea or interferon-α for approximately 10 years. Because of hydroxyurea-related progressive leukocytosis and hepatosplenomegaly, a suspicion of MF was confirmed through BM biopsy, and therefore, the patient was treated with ruxolitinib. At that time, the JAK2V617F allele burden was 90.2% and *BCR::ABL1* was negative. Nevertheless, after 1 year of ruxolitinib therapy, which produced Ph chromatosome and *BCR::ABL1* fused gene, the patient was diagnosed with CML. Additionally, the patient not only had a *BCR::ABL1* fusion that was identified by FISH or PCR but was also Ph-positive when assessed by routine cytogenetic analysis. Thus, the diagnosis of CML was confirmed. Although the patient was first treated with the TKI, imatinib, the patient showed no improvement in symptoms and had a poor response to TKI. However, 6 months after the patient switched from imatinib to fermatinib, both WBC and the patient spleen significantly improved, but the hematologic remission was still not achieved, indicating the failure of the treatment, according to the evaluation criteria of response. The cause of treatment failure is unclear and may be related to myelofibrosis or primary drug resistance. Craig et al reported similar results that two of the five patients assessed never established complete normalization of blood counts in the limited follow-up period before death (at 4 and 6 months following identification of BCR–ABL1, respectively). Leukocytosis, thrombocytopenia, and anemia were the most common group-wide findings.

The presence of the BCR-ABL1 fusion gene leads to a CML phenotype, while mutations in the JAK2 gene are linked to MPN. So far, few studies have reported that *BCR::ABL1* rearrangement/Ph chromosome and JAK2 V617F mutation can coexist in CML patients and the *BCR::ABL1* translocation was suggested to be a secondary event in the JAK2-mutated clone (Table 2).^1–11^ Overall, about 23 clinical cases of coexistence of *BCR::ABL1* and JAK2V617F mutations have been reported and main clinical characteristics are reported in Table 2.^1–11^ The incidence of dual driver mutations is vastly unknown, since these patients usually exhibit an indolent clinical phenotype with a favorable outcome. Soderquist et al^1^ reported that the incidence of JAK2 V617F- *BCR::ABL1* varied by 0.4%. For a total of 23 patients, 10 patients were male, and 13 were female. The median age at initial diagnosis was 60 years (range 39–76 years). The mean time to detection of the second alteration was 11 years (range 3–18 years). In each case, an initial *BCR::ABL1* assay was negative. Noteworthy, 6 of the 23 patients progressed to myelofibrosis, raising the possibility that these patients are more prone to progression. Seventeen patients underwent cytoreductive treatment with hydroxyurea. The response to TKI therapy varied, but JAK2 V617F remained detectable in those patients who were tested during the disease progression. Although TKIs are very effective for CML, no other similarly active TKI can selectively target JAK2 V617F. At the latest documented follow-up, 16 patients are alive and 7 have died.

**Table 2:** Previous reports of JAK2 V617F+ MPN patients transformed to CML.

References, year	No. patients	Sex	Age at 1st D	Diagnosis*	Time to second Dx, years	Splenomegaly (cm)	WBC (×10^9^/L)	PLT (×10^9^/L)	HB (g/L)	BCR-ABL quantitation (PCR)	FISH BCR-ABL	Karyotype	JAK2 (% Allele frequency)	Bone marrowreticulin	Treatment	Alive/Dead
Soderquist et al, 2018^1^	1	F	66	ET	3	ND	ND	ND	ND							A
				Post-ET MF		ND	ND	ND	ND						Hy and Rux	
				CML		ND	42	38	10.2	Positive, e13a2	ND	46,XX,t(9;22)(q34;q11)	Positive, 24%		Nil, Hy and Rux	
Kandarpa et al, 2017^2^	2	M	63	Post-ET MF	2	3	5.4	496	13.3	Negative	Negative	ND	Positive	Moderate	Im	A
				CMF		18	24	575	13.3	4.40%	50%	ND	Positive,7%	MF-3	Das and Rux	
Kandarpa et al, 2017^2^	3	M	70	Post-ET MF	4	ND	3.8	589	14.3	No detected			ND	Moderate	Im	
				CMF		10	15.2	287	10.2	93.3% (IS)	ND	ND	CALR Positive	MF-3	Das and Rux	
Kandarpa et al, 2017^2^	4	F	59	ET	13	No	48.2	380	11.7	Negative	ND	ND	Positive	Mild to none	Im	A
				CML		No	9.7	383	8	3.291% (IS)	ND	ND	Positive	MF-3	Das and Rux	
Grisouard et al, 2013^5^	5	F	51	ET	5	ND	30	ND	ND	ND	ND	46,XX,t(9;22)(q34;q11)	ND	ND	Anagrelide	
				CML		ND	ND	ND	ND	Positive, b3a2	ND	t(9;22)(q34;q11)	ND	ND	Hy and Das	
Jallades et al, 2008^4^	6	F	56	ET	4		7.5	669	13.5							A
				CML		16.7	100~	1000	13.5		81%	,t(9;22)(q34;q11)	positive		Im	
Curtin et al, 2005^6^	7	M	73	ET	12	13.3	108	790	15.3		Negative	46, XY	ND	No fibrosis	Aspirin	A
				CML			40.6	365	ND		67%	46,XY,t(9;22)(q34;q11)	ND	ND	Hy or Im	
Wahlin and Golovleva, 2003^7^	8	M	41	ET	18	Normal	Normal	1108	Normal			46, XY	2.65	Normal	Hy and IFN	A
				CML			60.1	ND	DN	ND	99.5%	46,XY,t(9;22)(q34;q11)	93.5%		Peripheral SCT	
Soderquist et al, 2018^1^	9	F	48	PV	5										Phleb, Hy, IFN	A
				Post-pv MF											IFN and Rux	
				CML			26.1	66	13.4	e13a2	ND	46,XX,t(9;22)(q34;q11)	24.4%		Im, IFN and Rux	
Soderquist et al, 2018^1^	10	F	60	PV	9										Hy	D
				Post-pv MF											Th or Rux	
				CML			60.2	77	8.8	e1a2	63%	46,XX,t(9;22)(q34;q11)	>50%		Im, Th and Rux	
Soderquist et al, 2018^1^	11	M	76	PV	6										Hy	D
				CML (AP)			233	82	10	e1a2	82%	46,XY,t(9;22)(q34;q11)	<10%		Hy or Im	
Kandarpa et al, 2017^2^	12	F	68	Post-PV MF	15	18.1	93	334	109	Negative			Positive	Moderate	Im	A
				CML		16	ND	ND	ND	ND	11%	ND	Positive	High	Das and Rux	
Zhou et al, 2015^8^	13	F	45	PV	11											
				CML		Yes	45	799	95			46,XX,t(9;22)(q34;q11)	6%		Das, Hy and Rux	
Wang et al, 2013^9^	14			PV	12					Positive			Positive		Hy	A
				CML			45	ND	95	Positive			Positive			
Wang et al, 2013^9^	15	ND	ND	PV	18					Positive			Positive			A
				CML						Positive			Positive			
Pieri et al, 2011^10^	16	M	72	PV	10										Hy	
				CML			46	462	118	b3a2		46,XY,t(9;22)(q34;q11)	61%		Im and Das	
Pingali et al, 2009^12^	17	M	39	PV	15										Phleb	A
				CML		8	662	342	129			t(9;22)(q34;q112)	Positive		Im, Nil and Das	
Hussein et al, 2008^13^	18	M	48	PV	15	16.7	100~	1000	135	ND	81%	46,XY,t(9;22)(q34;q11)	62%			D
				CML						b3a2			75%		Im and IFN	
Bocchia et al, 2007^14^	19	M	43	PV	16						t(9;18)(p10;q10)	9p+	Positive		Phleb	**A**
				CML		Yes	100					46,XX,t(9;22)(q34;q11)/der(t9;18)(p10;q10)	27%		Im	
Mirza et al, 2007^3^	20	F	66	PV	16										32P‡ and Hy	**D**
				CML		Yes	677	605	98		60%	t(9;22)(q34;q112)	Positive		Hy and Im	
Mirza et al, 2007^3^	21	F	48	PV	15										Phleb and Hy	**A**
				CML		25	102	65	2144			t(9;22)(q34;q112)	Positive		Im	
Soderquist et al, 2018^1^	22	F	48	PMF	10										Without therapy	**A**
				CML			142	557	83	e13a2	94%	46,XX,t(9;22)(q34;q11)	10-50%		Nil, Hy and Rux	
Yamada et al, 2014^11^	23	M	67	PMF	3		22.6	652	90~			Normal	Positive		Without therapy	**A**
				CML		Yes					90%	t(9;22;21)(q34;q11;q22)	Positive		Nil and Das	

Abbreviations: A = alive, AP = accelerated phase, CML = chronic myeloid leukemia, D = dead, Dx = diagnosis, ET = essential thrombocythemia, F = female, FISH = fluorescence in situ hybridization, HB = hemoglobin, Hy = hydroxyurea, IFN = interferon-α, Im = imatinib, M = male, ND = no descriptive, ND = not determined, Nil = nilotinib, Phleb = therapeutic phlebotomy, PLT = platelet, PMF = primary myelofibrosis, PV = polycythemia vera, Rux = ruxolitinib, SCT = stem cell transplantation, Th = thalidomide, WBC = white blood cell.

* The disease diagnosed first or is more prominent is listed first.

Many reports have questioned the clonal composition of MPNs, harboring both BCR-ABL1 and JAK2 V617F. BCR–ABL1 and JAK2 V617F can exist in completely independent clones or in the same clone, but with the majority of cases likely reflecting the presence of unrelated and independent clones.^1^ In addition, we detected several mutations by NGS, and we did find nondriver mutations, including IDH2 (44.90%), ASXL1 (2.1%), KRAS (5.8%), RUNX1 (4.5%), and IDH1 (1.9%). The relative high frequency of these mutations in our series suggests that there is no dominance of MF in later development of CML, which can explain the presence of 2 diseases in the same patient. In addition, the presence of these mutations indicates poor prognosis in MF and suggest short overall survival. How to explain the transformation of MPN to CML. Is it clonal evolution or therapy-related CML? Therapy-related CML has been reported in other cancers, including breast, lung, and gastric cancers, but very little is known about its clinical presentation and pathologic features.^15^ Although treatment strategies would be exactly the same as those for de novo CML, little is known about the responses and outcomes of therapy-related CML patients treated with TKIs. Iriyama et al^15^ reported 11 patients with therapy-related CML treated with TKIs. The responses, prognoses, treatment responses, and outcomes were favorable as those of patients with de novo CML.^15^ Although therapy-related CML have been reported to be potentially related to chemotherapy, radiotherapy, and immunosuppressive therapy, the pathogenesis of therapy-related CML is unclear. Either chemotherapy or radiotherapy could have had their immunity aggrieved or BM microenvironment injured.

Interestingly, low levels of *BCR::ABL1* transcripts have been detected in some MPN at the cytogenetic level.^16,17^ This and the chronology of reported transformations of PV or ET to CML suggest that the emergence of CML is likely a secondary event that results in the expansion of a clone with a greater proliferative advantage. It also appears to be consistent with the epidemiologic data that have shown that the pathogenesis of chronic phase CML is a result of 2 or more genetic events rather than a single hit.^[18]^ However, others have suggested the mutations may arise in two independent clones. Nevertheless, both these scenarios presuppose an unstable genome that induces multiple changes in a stem cell or favors emergence of other competing clones. However, another hypothesis suggests that JAK2 V617F mutation increases genetic instability, resulting in the acquisition of a translocation or loss of heterozygosity. Alternatively, the patient may have a germline predisposition to leukemia, or a JAK2 V617F mutation that may have occurred in an HSC together with other somatic mutations that induce genetic instability.^[11]^

In conclusion, we describe a rare case of a patient who was diagnosed with ET followed by a diagnosis of CML and demonstrated that a preexisting JAK2 V617F positive clone acquired *BCR::ABL1* translocation. This might suggest the necessity of screening for *BCR::ABL1* translocation in patients with MPN with poor treatment responses and a rapid megalosplenia.
